# The impact of different intervention methods (anesthetic adjuvant drugs, anesthetic techniques, and non-pharmacological interventions) on postoperative cognitive function in patients undergoing cardiac surgery: a systematic review and Bayesian network meta-analysis

**DOI:** 10.3389/fneur.2026.1814218

**Published:** 2026-07-24

**Authors:** Jiao Xue, Xiaodong Wang, Yi Qiu

**Affiliations:** Department of Anesthesiology Center, The Second Affiliated Hospital of Inner Mongolia Medical University, Hohhot, Nei Mongol, China

**Keywords:** cardiac surgery, general anesthesia, network meta-analysis, postoperative cognitive dysfunction, postoperative delirium

## Abstract

**Objective:**

Postoperative cognitive dysfunction (POCD) is a frequent complication after cardiac surgery, with inconsistent evidence regarding preventive strategies. This network meta-analysis aims to evaluate and compare multiple interventions to establish an evidence-based framework for optimizing clinical management.

**Methods:**

A systematic search of PubMed, Embase, the Cochrane Library, and Web of Science was conducted for randomized controlled trials (RCTs) involving patients undergoing cardiac surgery. The study, adhering to PRISMA-NMA guidelines, included various interventions such as anesthetic agents, adjunctive drugs, and cognitive training. Methodological quality was assessed using the Cochrane Risk of Bias tool. We performed a Bayesian network meta-analysis. Rankings were interpreted according to the prespecified favorable outcome direction; therefore, lower SUCRA values denoted greater preventive effectiveness.

**Results:**

The analysis included 46 RCTs, encompassing 9,928 patients and 20 different intervention strategies. Most studies demonstrated a low risk of bias. Because POD and POCD were adverse-event incidence outcomes and MMSE change was coded so that smaller unfavorable postoperative decline represented benefit, lower SUCRA values were interpreted as indicating greater intervention effectiveness. Intranasal insulin, gastrodin, and cognitive training showed the lowest SUCRA values for POD-related outcomes. Gastrodin, esketamine, and dexmedetomidine showed the lowest SUCRA values for POCD-related outcomes. MMSE-change rankings were interpreted cautiously because they were based on fewer studies and were sensitive to the coding direction of change scores.

**Conclusion:**

This network meta-analysis suggests that intranasal insulin, gastrodin, cognitive training, esketamine, and dexmedetomidine may reduce POD or POCD after cardiac surgery. However, the comparative rankings are uncertain for interventions supported by few studies, and the MMSE-change findings remain exploratory. Further high-quality RCTs are required to confirm these results.

**Systematic review registration:**

https://www.crd.york.ac.uk/PROSPERO/view/CRD420251128365, identifier (CRD420251128365).

## 1 Introduction

In 2018, an international working group recommended perioperative neurocognitive disorders as an overarching term for cognitive changes associated with surgery and anesthesia. The framework includes pre-existing neurocognitive disorder, postoperative delirium within 7 days, delayed neurocognitive recovery within 30 days, and postoperative neurocognitive disorder from 30 days to 12 months after surgery [1]. Because the included trials used heterogeneous historical definitions, the term POCD is retained in this review to reflect the terminology reported by the original studies. Despite significant advancements in surgical techniques, anesthesia management, and perioperative care that have markedly improved patient safety, surgery and anesthesia continue to pose risks of both structural and functional injury to the central nervous system [2]. POD is one of the most common yet frequently underdiagnosed complications, particularly among elderly patients undergoing general anesthesia. General anesthetics can influence the central nervous system by altering neurotransmission and suppressing cortical activity. Clinical manifestations of POD include impaired attention, disorientation, perceptual disturbances, and behavioral changes. However, atypical presentation, limited physician awareness, lack of standardized screening tools, and fluctuating symptoms make POD difficult to identify [3]. These diagnostic difficulties often delay timely recognition and treatment [4]. Previous studies have reported that the incidence of postoperative delirium ranges from 26 to 52%, while the incidence of POCD ranges from 35 to 60% [5]. The development of POD significantly diminishes patients’ postoperative quality of life, increases the burden on healthcare providers, and contributes to elevated healthcare costs. Affected patients face a heightened risk of complications, including aspiration, nosocomial pneumonia, pulmonary embolism, and pressure injuries. In mechanically ventilated patients, POD may lead to unplanned extubation, necessitating reintubation or delayed extubation, which prolongs hospital stays, escalates medical expenditures, and increases both ICU readmission and mortality rates [6]. Furthermore, POD is linked to persistent cognitive decline and serves as a critical risk factor for the development of POCD within the first month following surgery [7]. A multicenter cohort study by Kirfel et al. reported that elderly patients undergoing coronary artery bypass grafting (CABG) with cardiopulmonary bypass (CPB) experienced a POCD incidence of nearly 60% within seven days postoperatively, which is substantially higher than that observed in patients undergoing non-cardiac surgery [3]. Several intraoperative factors contribute to this elevated risk, including systemic and cerebral hypoperfusion, microemboli formation during CPB and aortic manipulation, rapid temperature fluctuations, and systemic inflammatory response syndrome (SIRS). These mechanisms can result in cerebral metabolic disturbances, neuroinflammation, and neurotransmitter imbalances, which together underlie the high incidence of POD following cardiac surgery [8]. Preventing POD and POCD after cardiac surgery remains an urgent clinical challenge. In recent years, both pharmacological and non-pharmacological interventions have been explored to mitigate these complications. Among pharmacological approaches, agents such as esketamine, ifenprodil, mannitol, and dexamethasone have demonstrated efficacy in reducing the incidence of POD and POCD through mechanisms including sedation, anti-inflammatory effects, and neuroprotection [9]. Other drugs, such as intranasal insulin, valerian, lidocaine, and erythromycin, appear to attenuate postoperative neuroinflammation by enhancing cerebral glucose metabolism, improving neuronal function, and providing analgesic and anti-inflammatory benefits, thereby demonstrating potential in alleviating cognitive dysfunction. Additionally, dexmedetomidine and propofol have been investigated for their potential roles in preserving postoperative cognitive function [10]. Sevoflurane has also been evaluated as part of anesthetic strategy comparisons in this field [11]. Compared with pharmacological approaches, non-pharmacological interventions are more diverse and include strategies such as remote ischemic preconditioning (RIPC), music therapy, family involvement, psychological support, cognitive training, and ABCDE bundled care. These interventions are generally low-cost, easy to implement, and offer notable advantages in terms of safety [12]. Nevertheless, the existing literature is extensive and often presents conflicting findings. Intervention practices aimed at preventing POD and POCD vary significantly across institutions and lack standardized methodologies, which leaves the optimal approach uncertain. Consequently, this study employs a network meta-analysis to systematically compare the effects of various intervention strategies on postoperative cognitive function, aiming to provide evidence-based guidance for optimizing clinical practice.

Bayesian network meta-analysis, as an advanced form of systematic review, facilitates the simultaneous comparison of both direct and indirect evidence across multiple interventions, providing reliable rankings of effectiveness even in the absence of head-to-head clinical trials. Consequently, the objectives of this study are: (1) to systematically review the primary interventions currently employed in patients undergoing cardiac surgery under general anesthesia for the prevention or mitigation of POD and POCD; (2) to utilize Bayesian network meta-analysis to comprehensively evaluate the relative effects of these interventions on postoperative delirium and cognitive function; and (3) to identify the most effective intervention strategies, thereby offering evidence-based guidance to optimize perioperative management and reduce the incidence of POD and POCD.

## 2 Methods

### 2.1 Study design

This study was previously registered with the International Registry for Prospective Systematic Reviews (PROSPERO) (registration number: CRD420251128365).

### 2.2 Search strategy

Two researchers (XJ and QY) independently searched the PubMed, Web of Science, Embase, and Cochrane Library databases using the following keywords: cardiac surgery, general anesthesia, POCD, and POD. The interventions included in the search were: anesthetic adjuvants, anesthetic techniques, non-pharmacological interventions, and neuroprotective measures. To ensure the comprehensiveness of the search, the MeSH vocabulary was used in PubMed to expand the search scope. The search scope covered titles, abstracts, and keywords, with the study type limited to RCTs, and no restrictions on language or publication date. The search timeframe was from the establishment of the database to July 10, 2025. The systematic review was conducted strictly in accordance with the PRISMA guidelines [13].

### 2.3 Inclusion and exclusion criteria

Inclusion criteria:

1. Adult patients (≥18 years) undergoing cardiac surgery under general anesthesia;
2. RCTs;
3. Anesthetic agents (propofol, sevoflurane, esketamine, etc.); anesthetic adjuvants (dexmedetomidine, gastrodin, insulin nasal spray, etc.); neuroprotective measures (valerian, magnesium agents, RIPC, etc.); adjustments to anesthetic techniques or strategies; Non-pharmacological interventions (cognitive training, music therapy, acupuncture, electrical stimulation, exercise training, sleep management, virtual reality, etc.);
4. Outcome measures: Report at least one of the following cognitive-related outcomes: incidence of POD; incidence of POCD; cognitive function scores (e.g., MMSE, MoCA, etc.);
5. Both Chinese and English literature;

Exclusion criteria:

1. Non-randomized controlled trials (such as cohort studies, case–control studies, case series, reviews, conference abstracts, etc.);
2. Studies involving non-adults (<18 years old), animal experiments, or *in vitro* experiments;
3. Intervention measures that do not meet inclusion criteria or cannot be compared with other studies;
4. Studies that do not report relevant outcome measures such as POCD, POD, or cognitive function scores;
5. Duplicate publications (if multiple studies are based on the same data, only the most recent or most comprehensive study will be included).

### 2.4 Literature screening and data extraction

Literature from PubMed, Embase, the Cochrane Library, and Web of Science was managed using EndNote. Two reviewers independently screened titles and abstracts, assessed full texts, and resolved disagreements through discussion with a third reviewer. Data were extracted independently using a standardized form and cross-checked for accuracy. Extracted information included study characteristics, patient baseline data, inclusion criteria, and outcome measures. Primary outcomes were the incidence of postoperative delirium and postoperative cognitive dysfunction, while secondary outcomes included cognitive function scores such as the Mini-Mental State Examination.

To address potential confounding from routine anesthetic drugs, intervention nodes were defined according to the randomized intervention actually assigned in each trial. Propofol was coded as a separate intervention node only when it represented the randomized main maintenance anesthetic, postoperative sedation strategy, or explicit comparator; when propofol was used merely as a shared induction/background drug across trial arms, it was not interpreted as an isolated anti-delirium intervention.

### 2.5 Risk of bias assessments

The methodological quality of the included studies was independently evaluated by two reviewers using the Cochrane Risk of Bias tool (RoB 1). The assessed domains were random sequence generation, allocation concealment, blinding of participants and personnel, blinding of outcome assessment, incomplete outcome data, selective reporting, and other bias. Each domain was judged as low, unclear, or high risk. Disagreements were resolved through discussion with a third reviewer.

### 2.6 Statistical analysis

Statistical analyses were conducted in R Studio using the gemtc and BUGSnet packages to perform Bayesian random-effects network meta-analyses. Relative risks (RRs) with 95% CrIs were used for binary outcomes, and mean differences (MDs) with 95% CrIs for continuous outcomes. Bayesian Markov Chain Monte Carlo methods were used, with convergence assessed by the Gelman–Rubin diagnostic, where values close to 1 indicated adequate convergence. Inconsistency between direct and indirect evidence was evaluated using node-splitting analysis, with *p* > 0.05 indicating no significant inconsistency. Intervention efficacy was ranked using surface under the cumulative ranking curve values. Rank plots were generated from the software output, whereas all narrative rankings were interpreted according to the prespecified favorable outcome direction: for adverse incidence outcomes (POD and POCD), lower event risk was preferable; for MMSE change, smaller unfavorable postoperative decline was preferable. Therefore, lower SUCRA values denote greater preventive effectiveness throughout this manuscript.

## 3 Results

### 3.1 Literature search results

**Figure 1:**
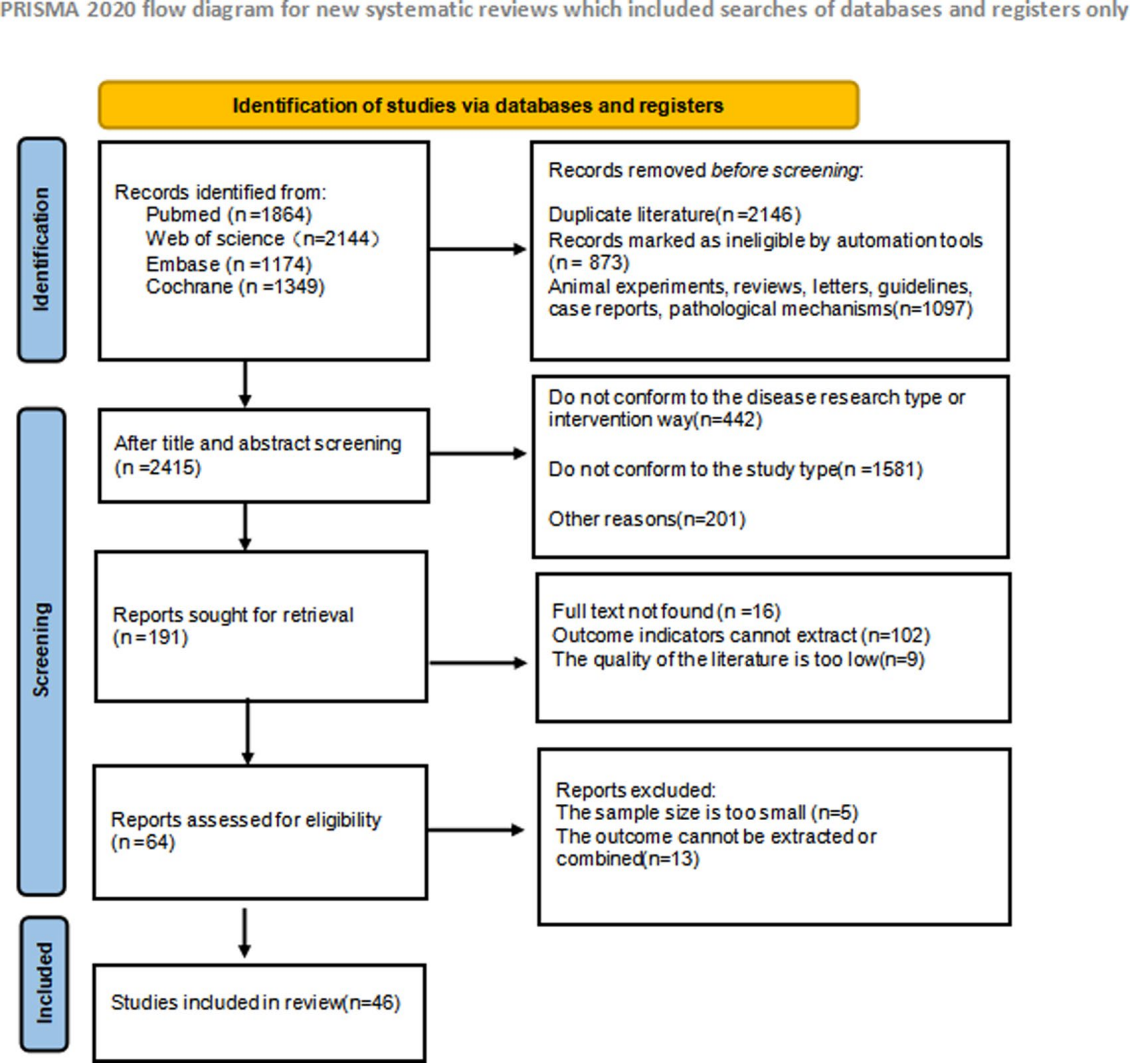
Literature screening process.

The database search identified 6,531 records. After removing 4,116 duplicates and ineligible publications, 2,415 articles remained. Title and abstract screening excluded 2,224 studies, leaving 191 articles for full-text review. Of these, 145 were excluded due to unavailable full texts, insufficient data, low methodological quality, or ineligible outcomes. Ultimately, 46 randomized controlled trials met the inclusion criteria and were included in the network meta-analysis. The study selection process is shown in Figure 1.

### 3.2 Basic characteristics of the included studies

As shown in Table 1, all 46 included studies were RCTs, involving a total of 9,928 adult patients undergoing cardiac surgery under general anesthesia, including 4,956 in the experimental group and 4,972 in the control group. The 46 randomized controlled trials included in this network meta-analysis are listed as references [14–59] and summarized in Table 1. The characteristics of the included studies, patient baseline information, and outcome measures are shown in Table 1.

**Table 1:** Clinical and demographic characteristics of studies included in NMA

Author	Year	Country	Study Type	Intervention Mode		Number of Cases		Age(years)		Gender (Male/Female)		Inclusion criteria	Outcome indicators
				Experimental group	Control group	Experimental group	Control group	Experimental group	Control group	Experimental group	Control group		
Yun-Xiao Bai [14]	2025	China	RCT	Gastrodin	Placebo	77	78	58.0 ± 8.5	58.3 ± 9.0	65\12	64\14	18–75 years old, elective coronary artery bypass grafting (CABG) ± valve replacement	POD, POCD, MMSE
Ming Yang [15]	2025	China	RCT	Intranasal insulin	Placebo	35	36	55.86 ± 5.65	55.92 ± 4.78	16\19	13\23	45–65 years old, elective extracorporeal heart surgery for middle-aged and young people (coronary artery bypass grafting, valve replacement, aortic surgery)	MMSE, POD
Liang-yu Ju [16]	2025	China	RCT	Esketamine	Placebo	67	67	65.0 ± 7.47	62.6 ± 8.32	54\13	49\18	Elective OPCAB, age ≥18 years, informed consent provided	POD
Qingqing Huang [17]	2024	China	RCT	Intranasal insulin	Placebo	36	35	53 (12)	55 (10)	12\24	14\21	Patients aged 18–65 years who underwent heart valve replacement surgery with a procedure that took 2–6 h under cardiopulmonary bypass (CPB);	POD
Yu Jiang [18]	2024	China	RCT	Cognitive training	Placebo	102	106	65 (58–70)	66 (58–70)	77\25	67\39	≥18 years old, elective coronary artery bypass grafting (CABG)	POCD, POD
Lingyu Lin [19]	2024	China	RCT	Cognitive training	Placebo	40	40	62.53 ± 7.83	59.55 ± 7.11	27\13	21\19	Patients after heart valve surgery	POD
Xinglong Xiong [20]	2024	China	RCT	Esketamine	Placebo	56	56	52.5(44.0–57.0)	51.5(47.2–56.0)	26\30	26\30	≥18 years old, ASA II/III, NYHA II/III, scheduled for open heart valve surgery with cardiopulmonary bypass	POD, MMSE
Carlos Méndez-Martínez [21]	2024	Spain	RCT	Cognitive training	Placebo	52	52	67.28 ± 10.55		23\29	40\12	Accepting elective heart surgery (aortic valve replacement, mitral valve replacement, coronary artery bypass grafting)	POD, POCD
Ayinoor V. Varsha [22]	2024	India	RCT	Propofol	Sevoflurane	36	36	59.34 ± 6.87	59.46 ± 10.08	30\4	28\7	Adult patients aged 18–80 years with coronary artery disease scheduled for elective coronary artery bypass graft surgery (CABG) with cardiopulmonary bypass (CPB).	POD
Masumeh Hemmati Maslakpak [23]	2024	Iran	RCT	Mannitol	Placebo	45	45	62.68±8.05	65.6±9.08	28\17	30\15	Patients aged 18–70 years scheduled for coronary artery bypass grafting	POD
Olivier Huet [24]	2024	France	RCT	Dexmedetomidine	Placebo	165	166	73 ± 5	73 ± 5	128\37	123\43	Patients aged 65 years or older who underwent elective cardiac surgery with or without cardiopulmonary bypass.	POD
Helena Claesson Lingehall [25]	2023	Sweden	RCT	Mannitol	Placebo	98	97	73 ± 4	73 ± 4	68\30	73\25	Patients aged 65 years or older listed for cardiac surgery at the Heart Centre of Umeå University Hospital	POD
Yosuke Nakadate [26]	2023	Japan	RCT	Intranasal insulin	Placebo	55	55	NA		NA		Atients undergoing scheduled cardiac surgery using CPB	POD
Shibiao Che [27]	2023	China	RCT	TTMP	Placebo	52	51	72.3 ± 7.1	71.5 ± 6.3	21\31	25\26	Patients undergoing elective heart valve replacement or coronary artery bypass surgery	POCD
Marius Butz [28]	2023	Germany	RCT	Cognitive training	Placebo	47	47	71.2 (4.7)	73.0 (4.9)	39\8	34\13	Elective aortic or mitral valve replacement/reconstruction with or without CABG under extracorporeal circulation and sufficient knowledge of German	POCD
Erica D. Wittwer [29]	2023	USA	RCT	Esketamine	Propofol	25	24	76.4 ± 3.5	76.1 ± 5.3	16\9	14\10	Age ≥70 years; undergoing complex cardiac surgery (more than one heart valve, redo sternotomy procedures, or combined valvular and CABG procedures)	POCD, POD, MMSE
Pia Vovk Racman [30]	2023	Slovenia	RCT	Dexmedetomidine	Propofol	37	34	83 (77–85)	83.5 (79–87)	17\20	17\17	Patients aged 18 or above who received TAVR via the femoral artery approach in the cardiology department.	MMSE, POCD, POD
Tingting Liu [31]	2023	China	RCT	RIPC	Placebo	225	223	47.6 ± 10.8	48.2 ± 11.3	132\93	134\89	18–80 years old; clear diagnosis requiring cardiovascular surgery	POD
Jia-Li Jiang [32]	2023	China	RCT	Desflurane	Propofol	341	343	54.1 ± 11.0	53.4 ± 11.2	153\188	150\193	Adult patients (ages 18 years or older) undergoing elective on-pump cardiac valve surgery or combined valve with CABG surgeries.	POD
Grace E. Namirembe [33]	2023	USA	RCT	Dexmedetomidine	Placebo	160	177	68 (64, 74)		146\14	152\25	Patients aged 60 years or older who underwent cardiac surgery with planned cardiopulmonary bypass	POD
H.-B. Wang [34]	2023	China	RCT	Dexmedetomidine	Placebo	326	326	54.0 (11.8)	53.8 (12.6)	159\167	166\160	Consenting adults (aged ≥ 18 y) scheduled for open cardiac valve surgery with bypass	POD
Michał Kowalczyk [35]	2022	Poland	RCT	Dexmedetomidine	Placebo	23	23	67(10)	66(5)	NA		Age > 18 years, elective CABG surgery, ASA class II or III, ejection fraction ≥ 40%.	POD
Shruti Chitnis [36]	2022	Canada	RCT	Dexmedetomidine	Propofol	34	33	NA		30\4	30\3	Patients older than 75 undergoing CABG or aortic valve replacement (AVR)	POD
Sutira Siripoonyothai [37]	2021	Thailand	RCT	Esketamine	Propofol	32	32	NA		14\18	21\11	Patients undergoing cardiac surgery with CPB	POD
Sandro Glumac [38]	2021	Croatia	RCT	Dexamethasone	Placebo	54	62	67.4 (9.4)	67.1 (9.8)	43\11	49\13	Patients aged between 41 and 84 years scheduled for elective coronary artery bypass graft surgery, heart valve surgery or combined surgery	POCD、MMSE
Tuğba Onur [39]	2022	Turkey	RCT	FiO₂	Placebo	50	50	60.58 ± 8.07	60.38 ± 7.44	32\18	30\20	Patients aged 18–80 years old who were scheduled for isolated CABG surgery with an American Society of Anesthesiology (ASA) score of ≥III.	POD
Valery V. Likhvantsev [40]	2021	Russia	RCT	Dexmedetomidine	Placebo	84	85	62.6 ± 6.7	62.4 ± 7.2	62\22	60\25	Patients older than 45 years undergoing elective coronary artery bypass grafting (CABG) or valve surgery	POD
Alparslan Turan [41]	2020	USA	RCT	Dexmedetomidine	Placebo	400	398	63 (11)	62 (12)	266 \130	287\107	Patients aged 18–85 years scheduled for cardiac surgery with cardiopulmonary bypass and heart rates ≥50 beats per min.	POD
Hoda Shokri [42]	2020	Egypt	RCT	Dexmedetomidine	Clonidine	144	142	63.75 ± 3.29	64.38 ± 4.81	77 \67	62 \80	adult patients (60–70 years) with ASA II and III, scheduled for elective isolated CABG	POD
Zhou M [43]	2019	China	RCT	Dexmedetomidine	Placebo	39	39	22.8 ± 3.3	24.9 ± 4.8	17\19	18\20	Patients aged 60 to 80 who undergo heart valve replacement surgery with cardiopulmonary bypass	POCD
Brian P. O’Gara[44]	2019	USA	RCT	Cognitive training	Placebo	20	20	70 ± 6	69 ± 7	14\6	15\5	Age 60–90, ≥10 days before cardiac surgery, ≥high school education	POD、POCD
Rebecca Y [45]	2019	USA	RCT	Lidocaine	Placebo	211	209	67 ± 9.1	67 ± 9.5	151\60	160\49	Adults of at least 50 yr of age and scheduled to undergo coronary artery bypass grafting (CABG)	POCD
Balachundhar Subramaniam [46]	2019	US	RCT	Dexmedetomidine	Propofol	30	30	69 (63-74)	71 (64-79)	25\5	26\4	Patients aged60yearsor older undergoing coronary arteryby- pass graft surgery with or without aortic	POD
Patrick Meybohm [47]	2018	Germany	RCT	RIPC	Placebo	692	693	NA		NA		Patients aged 18 or above undergoing elective extracorporeal circulation heart surgery	POCD
Li X [48]	2017	China	RCT	Dexmedetomidine	Placebo	142	143	66.4 ± 5.4	67.5 ± 5.3	95\47	102\41	Elderly patients (age ≥ 60 years) scheduled to undergo elective coronary artery bypass graft and/or valve replacement surgery	POD
Colin F. Royse [49]	2017	USA	RCT	Methylprednisolone	Placebo	250	248	73.4 (10.5)	74.3 (9.3)	147\89	162\84	Age more than 18 yr, European System for Cardiac Risk Evaluation (EuroSCORE)	POD
Evanthia Thomaidou [50]	2017	Greece	RCT	Erythromycin	Placebo	19	21	62.6 ± 8	66.6 ± 8	18\1	19\2	Elective coronary artery bypass grafting (CABG) surgery	POCD
George Djaiani [51]	2016	Canada	RCT	Dexmedetomidine	Propofol	91	92	72.7 (6.4)	72.4 (6.2)	68\23	70\22	Patients ≥60 yr undergoing elective complex cardiac surgery or ≥70 yr undergoing isolated coronary revascularization or single-valve repair/replacement surgery with CPB;	POD
Judith A. Hudetz [52]	2015	USA	RCT	RIPC	Placebo	15	15	66 ± 6	65 ± 9	15\0	15\0	Men ≥ 55 years of age undergoing elective CABG, mitral or aortic valve repair or replacement	POD、POCD
Soghra Hassani [53]	2014	Iran	RCT	Valeriana officinalis	Placebo	31	30	66 (62–68)	66 (63.7–69)	17\14	19\11	Adult patients of both sexes, aged 30–70 years, scheduled for elective CABG surgery using CPB.	MMSE
Patrick Meybohm [54]	2013	Germany	RCT	RIPC	Placebo	90	90	70 (42–86)	68 (23–83)	69\21	77\13	Patients aged ≥18 years who were scheduled to undergo differing types of cardiac surgery in which a cardiopulmonary bypass would be used.	POCD
Joseph P. Mathew [55]	2013	USA	RCT	Magnesium	Placebo	198	191	68.6 (8.4)	68.4 (8.0)	144\54	125\66	Scheduled to undergo coronary artery bypass grafting, valve, or coronary artery bypass grafting plus valve surgery with CPB	POCD
C. F. Royse [56]	2011	Australia	RCT	Propofol	Desflurane	89	91	63.8 (10.6)	61.8 (10.4)	80\9	73\18	More than 18 years of age, undergoing primary elective coronary artery bypass surgery with cardiopulmonary bypass	POCD
Zhao Zhang [57]	2011	China	RCT	Gastrodin	Placebo	100	100	58±23	57±24	44\56	45\55	Adult patients (>18 years of age) undergoing mechanical mitral valve replacement due to mitral valve stenosis resulting from rheumatic fever	POCD
J. A. Hudetz [58]	2009	USA	RCT	Esketamine	Placebo	26	26	68 ± 7	67 ± 8	26\0	26\0	Elective coronary artery bypass graft surgery and/or valve replacement/repair procedures with a CPB	POCD
Antonino S. Rubino [59]	2010	Italy	RCT	clonidine	Placebo	15	15	63.9 ± 8.9	61.3 ± 6.3	10\5	8\7	Consecutive patients undergoing surgery for acute type-A aortic dissection	POD

### 3.3 The quality assessment of the included studies

**Figure 2:**
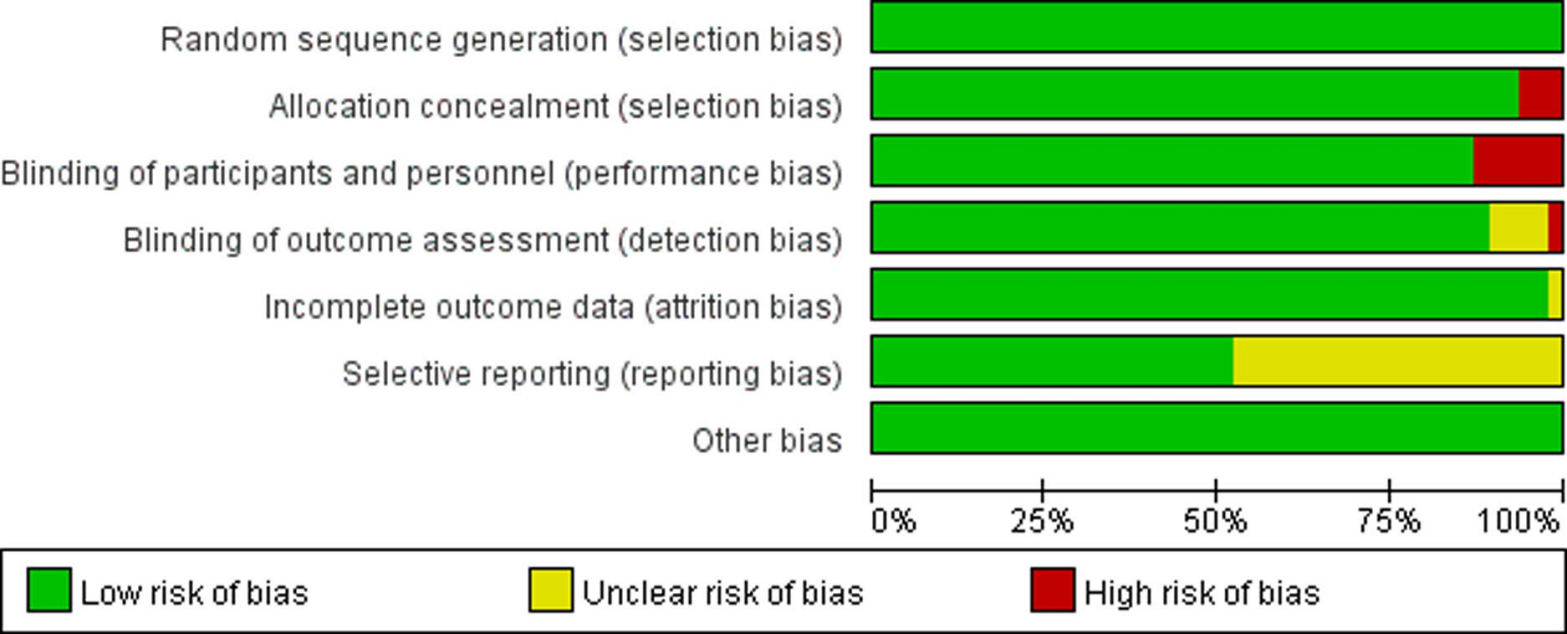
Risk of bias in randomized controlled trials.

The Cochrane Risk of Bias Tool (Review Manager 5.4) was used to assess methodological quality in 46 studies. Most trials showed low risk of bias in random sequence generation, outcome assessment blinding, completeness of outcome data, and other domains, indicating generally high methodological quality. However, concerns remained regarding allocation concealment, blinding of participants and personnel, and selective reporting. Several studies, including Rubino 2010, Méndez-Martínez 2024, Varsha 2024, Lin 2024, Racman 2023, and Yu Jiang 2024, exhibited high risk in allocation concealment or blinding. Inadequate reporting or implementation of allocation concealment may have introduced selection bias. Similarly, incomplete blinding increased the risk of performance bias. Additionally, some studies were judged to have unclear risk of selective reporting or outcome integrity due to limited methodological detail, lack of protocol registration, incomplete outcome reporting, or unclear handling of missing follow-up data (Figure 2).

### 3.4 Certainty of evidence

This study used GRADEpro to assess the certainty of the evidence. Table 2 summarizes the certainty of evidence for POD, POCD, and MMSE outcomes. The evidence suggested that the analyzed interventions were associated with reduced POD and POCD burden. The evidence for MMSE change was moderate and should be interpreted cautiously because the pooled estimate was close to the null. Overall, the certainty assessment supports the clinical relevance of interventions targeting POD and POCD after cardiac surgery.

**Table 2:** Certainty of evidence.

**Quality assessment**							**No of patients**		**Effect**		**Quality**	**Importance**
**No of studies**	**Design**	**Risk of bias**	**Inconsistency**	**Indirectness**	**Imprecision**	**Other considerations**	**Experimental**	**Control**	**Relative** **(95% CI)**	**Absolute**		
**POD**												
34	Randomized trials	No serious risk of bias	Serious	No serious indirectness	No serious imprecision	Reporting bias strong association	511/3392 (15.1%)	732/3409 (21.5%)	OR 0.54 (0.44–0.67)	86 fewer per 1000 (from 60 fewer to 107 fewer)	MODERATE	CRITICAL
								24.8%		97 fewer per 1000 (from 67 fewer to 121 fewer)		
**POCD**												
19	Randomized trials	No serious risk of bias	Serious	No serious indirectness	No serious imprecision	Reporting bias strong association	397/1853 (21.4%)	545/1856 (29.4%)	OR 0.61 (0.52–0.72)	91 fewer per 1000 (from 63 fewer to 116 fewer)	MODERATE	CRITICAL
								42%		114 fewer per 1000 (from 77 fewer to 146 fewer)		
**MMSE**												
8	Randomized trials	No serious risk of bias	Serious	No serious indirectness	No serious imprecision	Reporting bias strong association	427	432	–	MD 0.13 lower (0.37 lower to 0.1 higher)	MODERATE	SECONDARY

CI: Confidence Interval; MD: Mean Difference; OR: Odds Ratio.

## 4 Network meta-analysis

### 4.1 The incidence of POD

**Figure 3:**
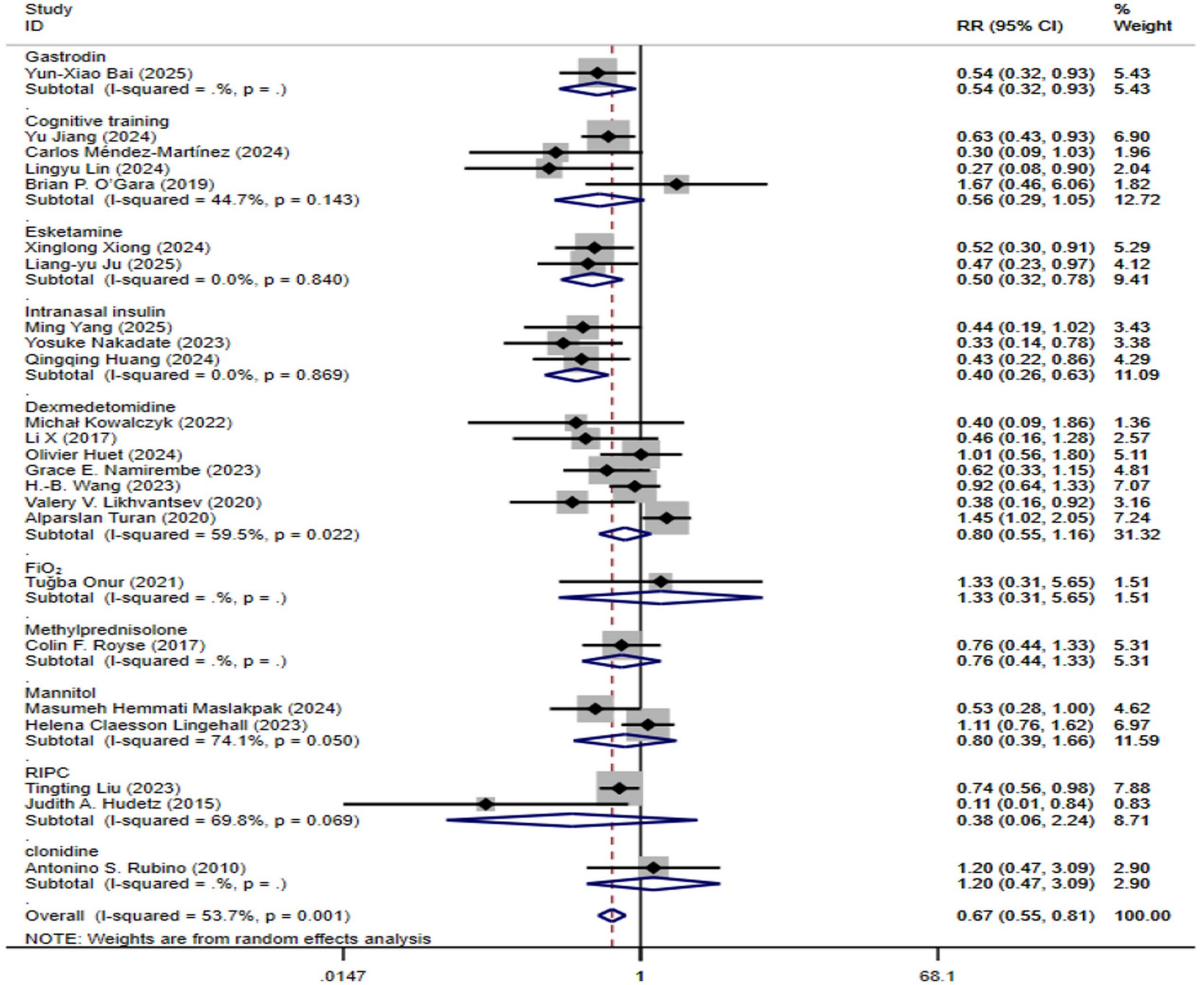
Forest plot of postoperative POD incidence in the experimental group and placebo group. RR: risk ratio.

A total of 24 placebo-controlled studies contributed to the conventional pairwise comparison, whereas 34 RCTs contributed to the POD network meta-analysis. Due to the large variability in intervention categories and significant heterogeneity among studies (*p* < 0.001, I^2^ = 53.7%), we used a random-effects model for the meta-analysis. The results showed (Figure 3) that different interventions had varying effects on reducing POD. Intranasal insulin, esketamine, and gastrodin demonstrated favorable effects, while cognitive training showed a beneficial trend that did not reach statistical significance. Dexmedetomidine, oxygen therapy, methylprednisolone, mannitol, ischemic preconditioning, and clonidine did not show clear benefits in the pairwise comparison, possibly due to single-study evidence or small sample sizes. The overall pairwise meta-analysis suggested that the interventions collectively reduced POD risk (RR = 0.67, 95% CI 0.55–0.81).

#### 4.1.1 Network evidence graph

**Figure 4:**
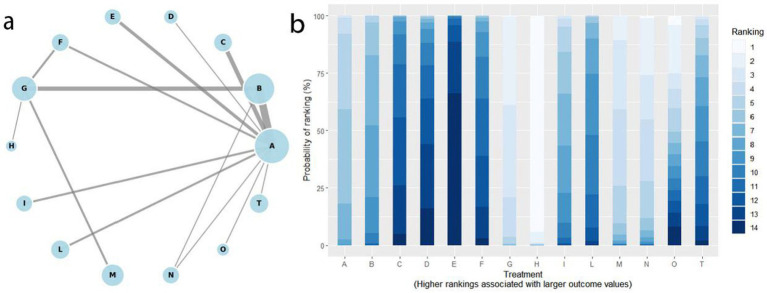
**(a)** Network evidence diagram; **(b)** cumulative ranking (SUCRA) diagram for POD. POD is an adverse incidence outcome; lower SUCRA values indicate greater preventive effectiveness in this analysis.

**Figure 5:**
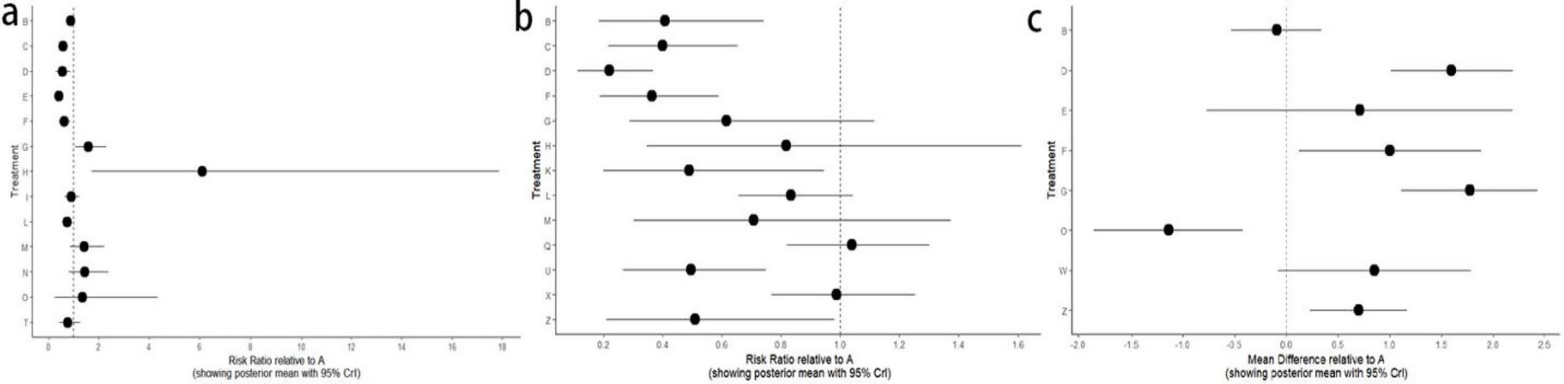
Forest plots comparing placebo (A) with each intervention: **(a)** POD, **(b)** POCD, and **(c)** MMSE change. Effect estimates are presented with 95% credible intervals.

**Figure6:**
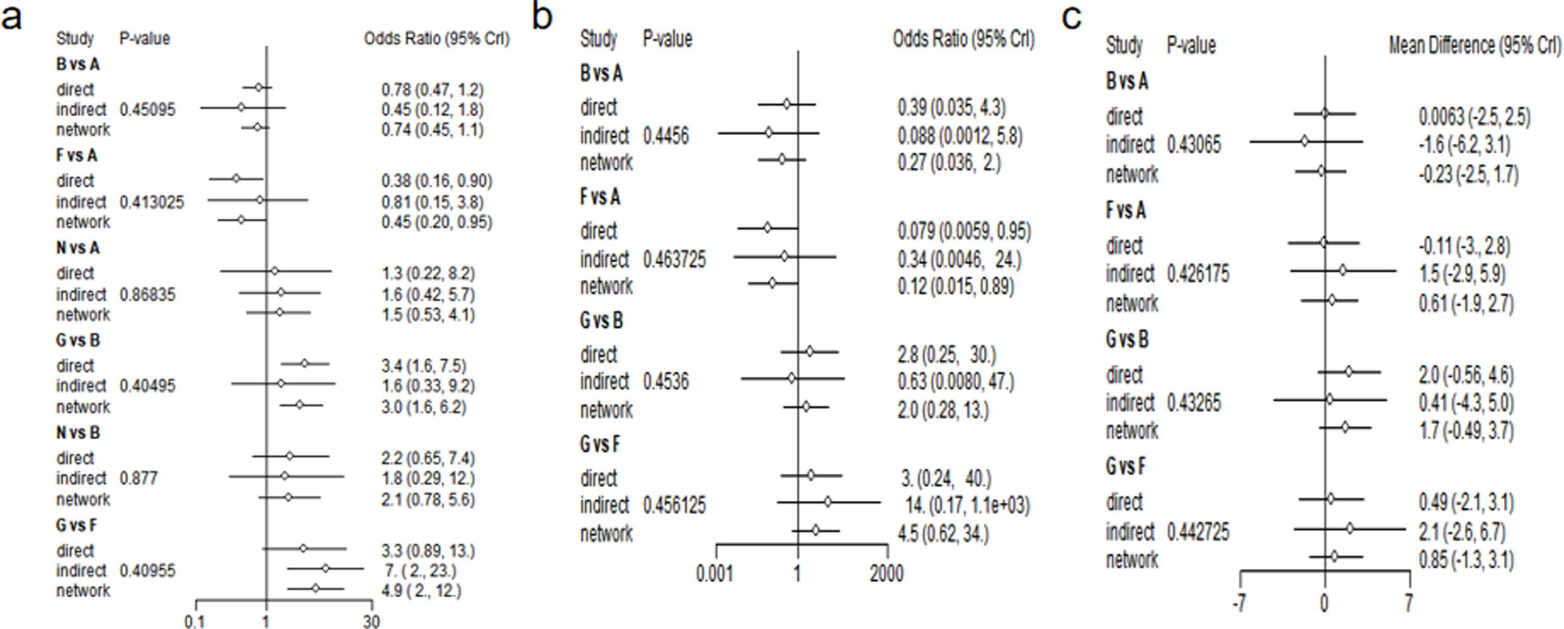
Node-splitting analyses for **(a)** POD, **(b)** POCD, and **(c)** MMSE change, showing direct and indirect estimates with corresponding 95% credible intervals and *p* values.

34 RCTs reported the incidence of postoperative POD, including 14 intervention measures: A: Placebo, B: Dexmedetomidine, C: Cognitive training, D: Gastrodin, E: Intranasal insulin, F: Esketamine, G: Propofol, H: Sevoflurane, I: Mannitol, L: Desflurane, M: FiO₂, N: Lidocaine, O: Methylprednisolone, T: Clonidine). The network relationships are centered around placebo treatment, with dots representing each intervention measure. A direct comparison is indicated by a line connecting two points, and the thickness of the line represents the number of included studies. The evidence network relationships are shown in Figure 4a. The forest plot comparing placebo with each intervention is shown in Figure 5a. Due to the presence of closed loops between interventions, the node splitting method was used to compare the consistency between direct and indirect evidence. The analysis of nodes in closed loops showed that there was no statistically significant difference between direct and indirect comparisons (*p* > 0.05) (Figure 6a).

#### 4.1.2 NMA

**Figure 7:**
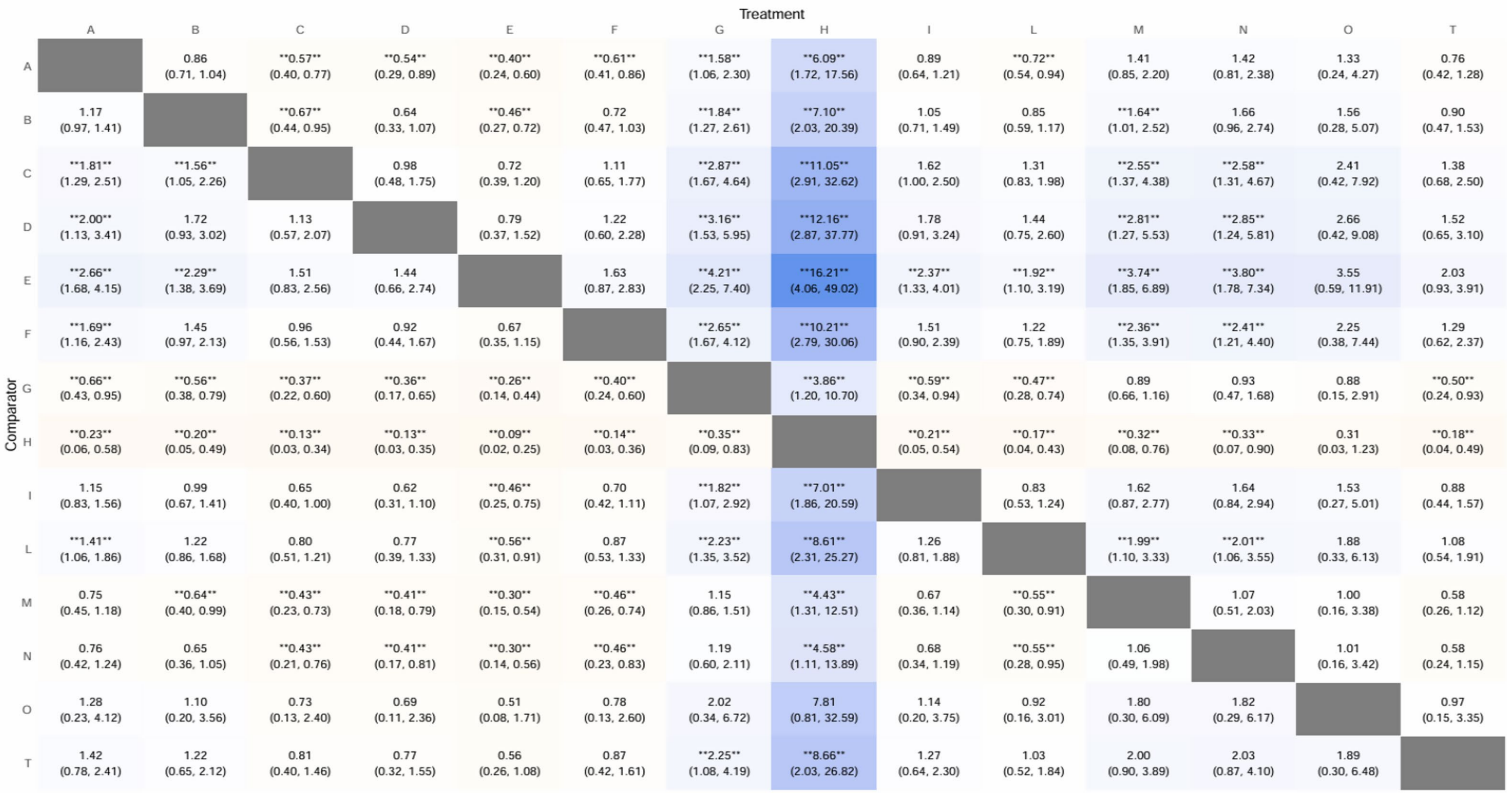
League table of the Bayesian network meta-analysis for postoperative delirium (POD). Cells show pairwise relative risks with 95% credible intervals. Bold values with asterisks indicate statistically significant differences; gray diagonal cells represent self-comparisons.

The network meta-analysis results showed 91 pairwise comparisons. The network analysis results for the incidence of POD are shown in Figure 7.

#### 4.1.3 Efficacy ranking

SUCRA probability ranking for POD showed: Intranasal insulin 3.87% < Gastrodin 16.42% < Cognitive training 19.15% < Esketamine 22.13% < Clonidine 33.79% < Lidocaine 37.72% = Methylprednisolone 37.72% < Dexmedetomidine 49.29% < Placebo 60.32% < FiO₂ 64.28% < Desflurane 77.84% < Mannitol 79.55% < Propofol 85.51% < Sevoflurane 99.57%. Because POD is an adverse incidence outcome, a lower SUCRA value indicates a better preventive effect against postoperative delirium. The SUCRA plot is shown in Figure 4b.

### 4.2 The incidence of POCD

#### 4.2.1 Network evidence graph

**Figure 8:**
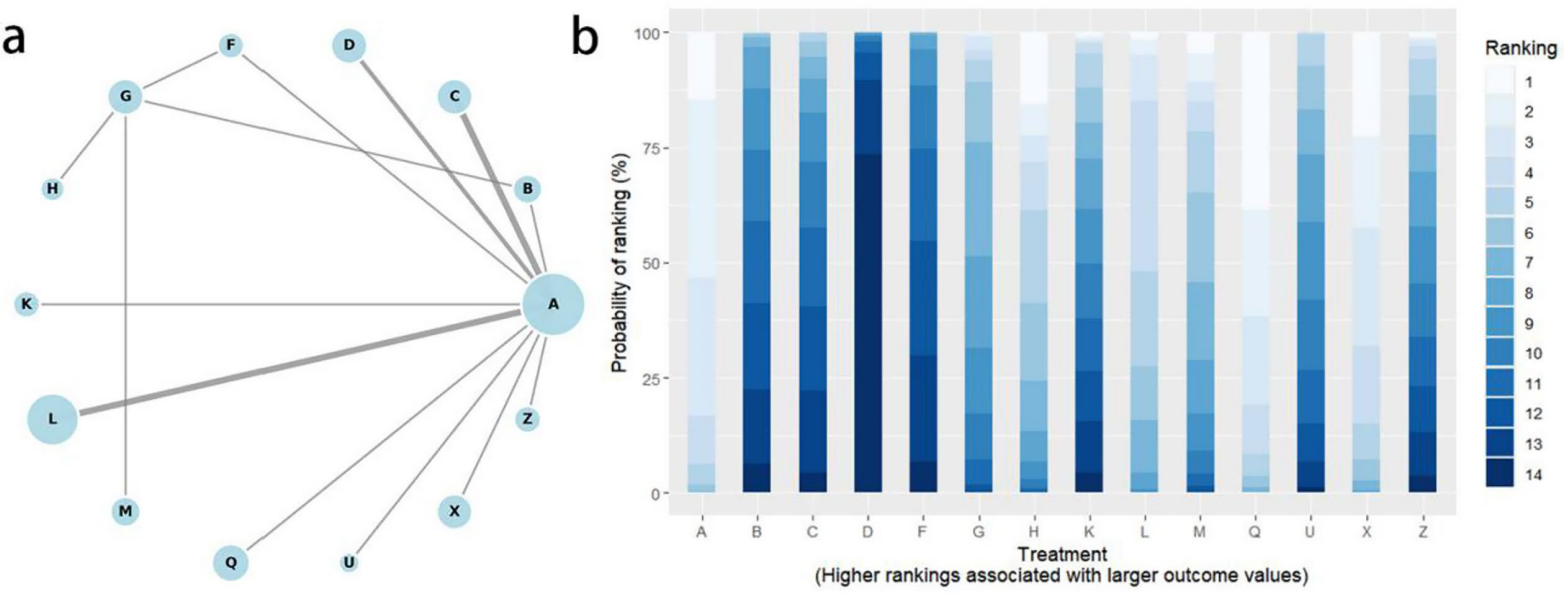
**(a)** Network evidence diagram; **(b)** Cumulative ranking SUCRA diagram for POCD. POCD is an adverse incidence outcome; lower SUCRA values indicate greater preventive effectiveness in this analysis.

19 RCTs reported the incidence of POCD, including 14 intervention measures (A: Placebo, B: Dexmedetomidine, C: Cognitive training, D: Gastrodin, F: Esketamine, G: Propofol, H: Sevoflurane, K: TTMP, L: Desflurane, M: FiO₂, Q: Lidocaine, U: Erythromycin, X: Magnesium, Z: Dexamethasone). The network relationships are shown in Figure 8a, and the forest plots comparing placebo with each intervention are shown in Figure 5b. Since there are closed loops between the interventions, the node splitting method was used to compare the consistency between direct and indirect evidence. The analysis results of the nodes in the closed loops showed that there was no statistically significant difference between the results of direct comparisons and indirect comparisons (*p* > 0.05) (Figure 6b).

#### 4.2.2 NMA

**Figure 9:**
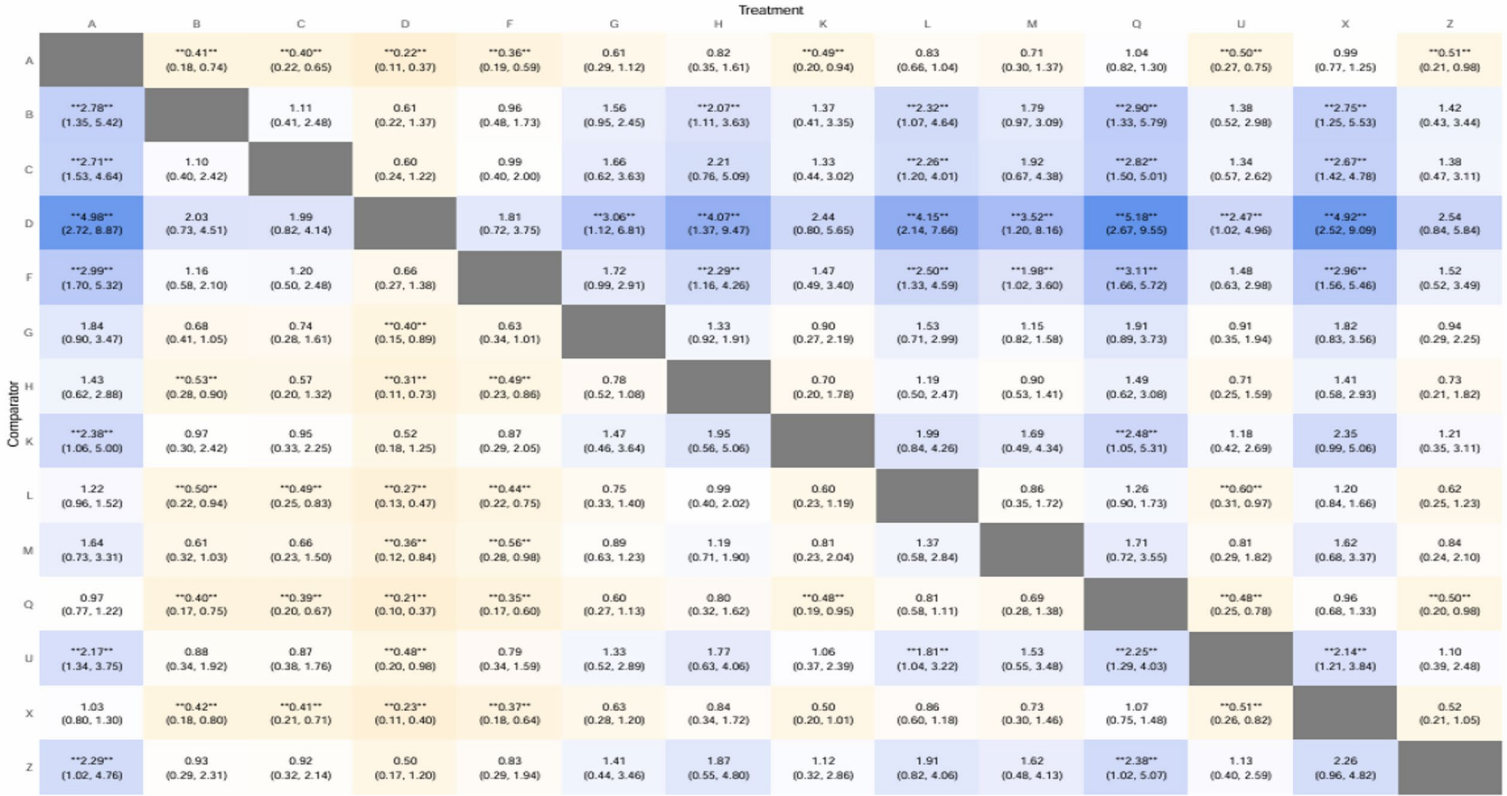
League table of the Bayesian network meta-analysis for postoperative cognitive dysfunction (POCD). Cells show pairwise relative risks with 95% credible intervals. Bold values with asterisks indicate statistically significant differences; gray diagonal cells represent self-comparisons.

The network meta-analysis results showed 91 pairwise comparisons. The network analysis results for the incidence of POCD are shown in Figure 9.

#### 4.2.3 Efficacy ranking

SUCRA probability ranking for POCD showed the most favorable values for Gastrodin (3.21%), Esketamine (18.39%), Dexmedetomidine (24.77%), and Cognitive training (26.65%). Erythromycin and Dexamethasone followed (35.64% each), followed by Lidocaine (35.94%), FiO₂ (64.21%), Desflurane (69.55%), Sevoflurane (74.94%), Magnesium and TTMP (83.55% each), and Placebo (88.11%); Propofol was not among the leading ranked interventions. Because POCD is an adverse incidence outcome, a lower SUCRA value indicates a smaller unfavorable postoperative event risk and therefore a better preventive effect against POCD. The SUCRA plot is shown in Figure 8b.

### 4.3 MMSE change

#### 4.3.1 Network evidence graph

**Figure 10:**
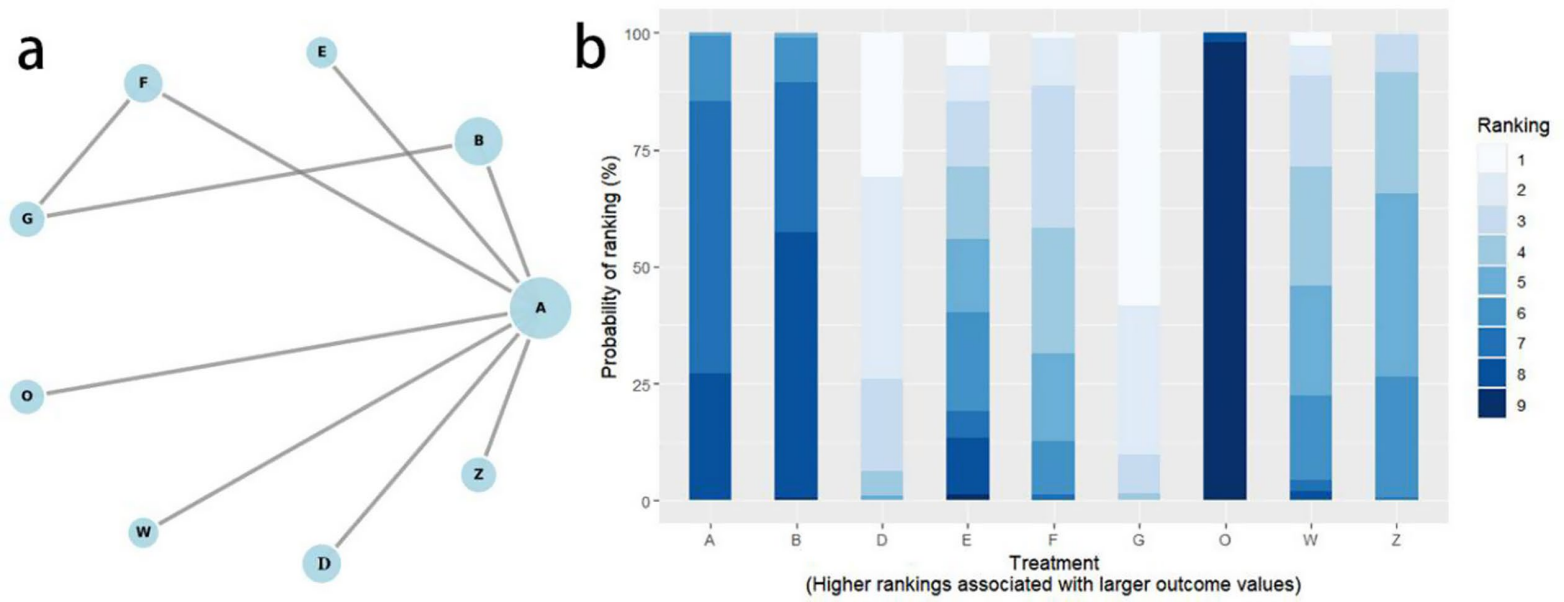
**(a)** Network evidence diagram; **(b)** Cumulative ranking SUCRA diagram for MMSE change. The MMSE-change score was coded so that smaller unfavorable postoperative decline represented benefit; lower SUCRA values therefore indicate greater effectiveness in this analysis.

Nine RCTs reported changes in MMSE scores, including nine treatment nodes (A: Placebo, B: Dexmedetomidine, D: Gastrodin, E: Intranasal insulin, F: Esketamine, G: Propofol, O: Methylprednisolone, W: *Valeriana officinalis*, Z: Dexamethasone). The network relationships are shown in Figure 10a, and the forest plots comparing placebo with each intervention are shown in Figure 5c. Since there are closed loops between the interventions, the node splitting method was used to compare the consistency between direct and indirect evidence. The analysis results of the closed-loop nodes showed that there was no statistically significant difference between direct and indirect comparisons (*p* > 0.05) (Figure 6c).

#### 4.3.2 NMA

**Figure 11:**
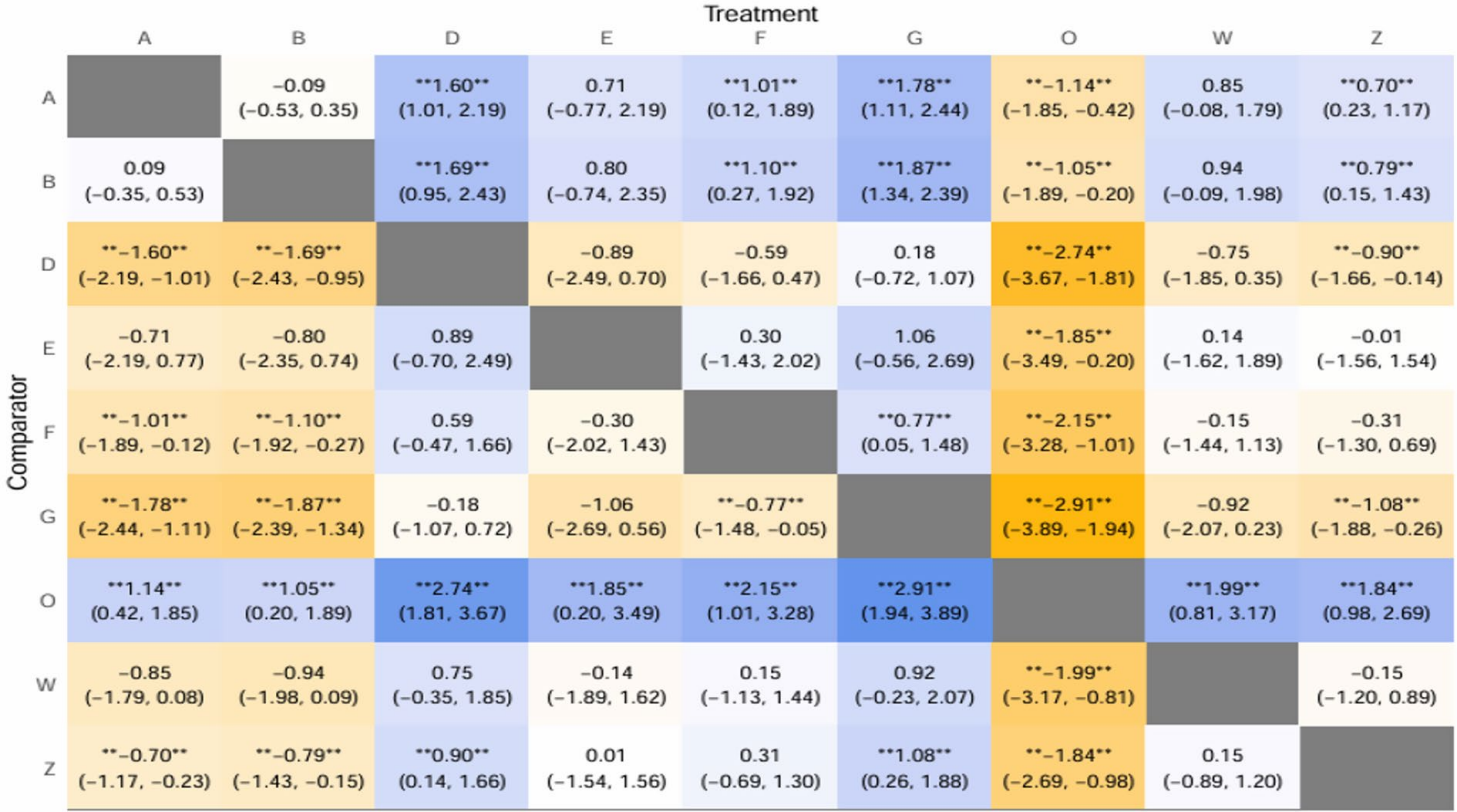
League table of the Bayesian network meta-analysis for MMSE change. Cells show pairwise mean differences with 95% credible intervals. Bold values with asterisks indicate statistically significant differences; gray diagonal cells represent self-comparisons.

The network meta-analysis results showed 36 pairwise comparisons. The network analysis results for MMSE changes are shown in Figure 11.

#### 4.3.3 Efficacy ranking

SUCRA probability ranking for MMSE change showed: Methylprednisolone 2.0% < Dexmedetomidine 19.2% < Placebo 23.5% < Dexamethasone 52.1% < Intranasal insulin 52.4% < *Valeriana officinalis* 58.1% < Esketamine 63.6% < Gastrodin 87.1% < Propofol 93.3%. Because the MMSE-change score was coded so that smaller unfavorable postoperative decline represented benefit, lower SUCRA values were interpreted as indicating greater effectiveness. However, because the MMSE network included fewer studies and the third-ranked treatment was placebo, this ranking should be interpreted cautiously rather than as definitive evidence of an active protective effect. The SUCRA plot is shown in Figure 10b.

### 4.4 Forest plots comparing placebo (A) with each intervention

Forest plot comparing each intervention measure with the placebo group in terms of various indicators (POD, POCD, MMSE) (Figure 5).

### 4.5 Heat map of network meta-analysis

**Figure12:**
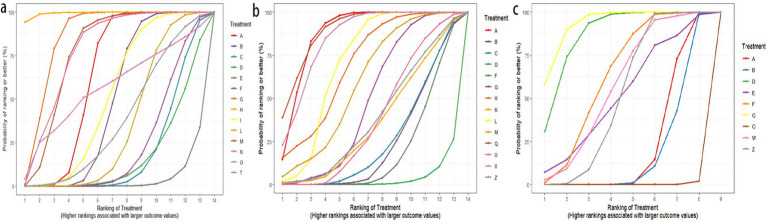
Cumulative ranking curves for **(a)** POD, **(b)** POCD, and **(c)** MMSE change. All rankings follow the prespecified direction in which lower SUCRA values indicate greater preventive effectiveness.

Differences in the performance of each treatment method in different rankings in POD, POCD, and MMSE are shown in Figure 12. All ranking interpretations follow the prespecified direction in which lower SUCRA values indicate better preventive effectiveness.

### 4.6 Node-splitting analysis

Closed loops were observed in POD, POCD, and MMSE. Therefore, a node analysis was employed for consistency testing, and the results showed that there were no significant differences in the direct and indirect comparisons of each outcome indicator (*p* > 0.05).

## 5 Discussion

The high incidence of postoperative neurocognitive dysfunction in cardiac surgery patients can be attributed to surgical complexity, anesthetic exposure, patient vulnerability, cardiopulmonary-bypass-related cerebral stress, and perioperative inflammation [60]. One major explanatory framework is cerebral metabolic insufficiency, in which perioperative hypoperfusion, embolic injury, and impaired autoregulation contribute to acute brain dysfunction [61]. A second framework is neuroinflammation, where systemic inflammatory activation disrupts the blood–brain barrier and alters neuronal excitability [62]. Clinical biomarker studies support this mechanism because elevated perioperative IL-6, TNF-alpha, and sTNFR-1 have been associated with a higher risk of POD after cardiac surgery [63]. The clinical importance of POD is substantial because delirium is associated with subsequent long-term cognitive decline [64]. Beyond cognitive outcomes, delirium also creates substantial social and economic burden in older patients [65].

In recent years, various pharmacological and non-pharmacological strategies have been investigated to prevent or alleviate POD and POCD in patients undergoing cardiac surgery, but their results remain inconsistent. Dexmedetomidine is one of the most frequently studied sedative adjuvants, and its potential benefit is usually attributed to cooperative sedation, opioid-sparing effects, sympathetic inhibition, and anti-inflammatory activity; however, the magnitude of benefit varies across studies and clinical contexts [66]. Volatile anesthetics remain controversial because small cardiac-surgery studies have reported different neurocognitive patterns after sevoflurane, desflurane, and isoflurane exposure [67]. Evidence from pediatric emergence research also indicates that changing from sevoflurane to desflurane does not necessarily translate into a lower delirium risk, suggesting that the effect of volatile anesthetics cannot be inferred from pharmacologic properties alone [68]. Cognitive training represents a different pathway because it aims to increase cognitive reserve before surgery rather than modify intraoperative neurochemistry [69]. Remote ischemic preconditioning may reduce postoperative brain injury by activating systemic protective signaling, but the pooled evidence in adult general-anesthesia populations remains unstable [70]. A cardiac-surgery-focused meta-analysis also reported inconsistent effects of remote ischemic preconditioning on POD and POCD, which supports a cautious interpretation of this intervention [71]. Strategies targeting cerebral oxygen delivery may be biologically plausible, but current evidence mainly supports monitoring and individualized prevention rather than a single fixed oxygen strategy [72].

The interpretation of propofol requires particular caution because propofol is commonly used as part of routine anesthetic practice rather than as a drug assigned specifically for delirium prevention. In cardiac anesthesia, propofol may appear as an induction agent, a maintenance anesthetic, an ICU sedative, or a comparator against volatile anesthesia or dexmedetomidine; these roles differ in exposure duration, depth-of-anesthesia effect, hemodynamic impact, and postoperative delirium assessment window. Therefore, in the present network meta-analysis, propofol was coded as an intervention node only when it represented the randomized main maintenance anesthetic, postoperative sedation regimen, or explicit comparator. When propofol was administered to both trial arms as a shared induction or background drug, it was not interpreted as an isolated anti-delirium intervention.

This distinction is important because a trial comparing propofol-based total intravenous anesthesia with sevoflurane in CABG patients evaluates a complete anesthetic strategy rather than the isolated molecular effect of propofol [22]. Similarly, the comparison of propofol with desflurane in coronary artery bypass surgery reflects a maintenance-anesthetic contrast, in which cerebral perfusion, emergence profile, anesthetic depth, and postoperative care may all contribute to cognitive outcomes [56]. In a postoperative sedation trial, propofol served as the comparator against dexmedetomidine, and the lower delirium rate in the dexmedetomidine group argues against treating propofol exposure itself as a clearly protective intervention [51]. A recent systematic review of cardiac-surgery RCTs found no clear neurocognitive advantage of propofol over volatile anesthetics, which further supports a conservative interpretation [73]. Accordingly, the propofol-related result in our ranking should be read as regimen-level evidence under specific trial designs, not as definitive proof that propofol alone prevents POD or POCD.

Esketamine also requires a balanced interpretation because its potential benefit and potential risk arise from closely related neuropharmacological actions. As the S-enantiomer of ketamine, esketamine antagonizes N-methyl-D-aspartate receptors and may reduce postoperative neurocognitive complications by improving analgesia, decreasing opioid exposure, attenuating central sensitization, and modulating inflammation. In cardiac valve surgery with cardiopulmonary bypass, Xiong et al. reported that esketamine reduced the risk of POD, suggesting that a carefully selected dose may be beneficial in a high-inflammatory-burden surgical setting [20]. In off-pump coronary artery bypass grafting, Ju et al. further reported that continuous intraoperative esketamine was associated with lower POD and a more favorable inflammatory response [16]. These cardiac-surgery findings are clinically plausible because pain, sleep disturbance, sympathetic activation, neuroinflammation, and cardiopulmonary-bypass-related cerebral stress all contribute to delirium risk.

At the same time, concerns about immediate post-anesthetic delirium are well founded. Ketamine-family drugs can produce dissociation, vivid dreams, hallucinations, emergence agitation, and psychotomimetic symptoms, and these manifestations may overlap with or complicate early delirium assessment. In the large PODCAST trial of older adults undergoing major surgery, intraoperative ketamine did not reduce postoperative delirium and was associated with more hallucinations and nightmares [74]. This negative signal explains why ketamine or esketamine has historically been viewed with caution in elderly or cognitively vulnerable patients during the immediate recovery period. A recent esketamine-focused meta-analysis suggested a possible reduction in POD, but it also emphasized heterogeneity in dose, timing, surgery type, and delirium measurement [75]. A broader ketamine/esketamine meta-analysis likewise indicated that conclusions are sensitive to study design and patient population [76]. The existence of an ongoing cardiovascular-surgery esketamine trial further shows that current evidence is still developing rather than definitive [77]. For this reason, esketamine should be described as a promising but still uncertain intervention that requires dose-specific evaluation, careful patient selection, and postoperative monitoring for neuropsychiatric adverse effects.

This study included 46 randomized controlled trials, covering 20 intervention measures and involving 9,928 patients who underwent cardiac surgery under general anesthesia. Because rankings were interpreted according to outcome favorability (lower POD/POCD incidence and smaller unfavorable MMSE change), lower SUCRA values were interpreted as indicating more favorable effects. For POD, intranasal insulin, gastrodin, and cognitive training had the lowest SUCRA values. For POCD, gastrodin, esketamine, and dexmedetomidine had the lowest SUCRA values. For MMSE change, however, the ranking was not treated as strong evidence for any active drug because placebo ranked among the leading treatments, indicating that the sparse MMSE network was unstable and sensitive to coding direction. Therefore, the clinical discussion focused primarily on POD and POCD, while MMSE was retained as an exploratory secondary outcome.

Intranasal insulin may be clinically relevant because perioperative brain insulin signaling is linked to glucose metabolism, synaptic function, and neuroinflammation. A randomized trial in elderly patients undergoing gastrointestinal surgery reported that repeated preoperative intranasal insulin reduced POD incidence, supporting the plausibility of this delivery route beyond cardiac surgery [78]. A comprehensive review also summarized potential cognitive effects of intranasal insulin in patients with diabetes, although the mechanisms and target populations remain heterogeneous [79]. Neuronal insulin receptor signaling provides a biological basis for these effects because it participates in synaptic plasticity and cellular survival pathways [80]. Intranasal administration can bypass the blood–brain barrier through olfactory and trigeminal pathways, which may explain why central effects can occur without high systemic exposure [81]. Neuroinflammation may still weaken cognitive resilience by disrupting BDNF/TrkB-related signaling and hippocampal function [82]. Experimental anesthesia models suggest that intranasal insulin can preserve synaptic plasticity and memory through mTOR-related pathways [83]. Another animal study linked the cognitive benefit of intranasal insulin to the mTOR-eEF2 pathway after anesthesia exposure [84]. Evidence from traumatic brain injury models also suggests that intranasal insulin may suppress inflammation and apoptosis through PI3K/Akt/mTOR signaling [85]. These mechanistic findings support the favorable POD ranking of intranasal insulin in our analysis, but clinical confirmation in cardiac-surgery populations remains necessary.

Gastrodin ranked favorably for POCD, but its evidence base should be interpreted with caution because the clinical data were mainly derived from a small number of trials. The recent randomized placebo-controlled cardiac-surgery trial by Bai et al. supports a potential effect on POD prevention [14]. The earlier double-blind trial by Zhang et al. suggested a possible benefit for cognitive decline after cardiopulmonary bypass, but the sample size was limited [57]. Mechanistically, gastrodin has anti-inflammatory activity, which may be relevant to cardiac-surgery-associated neuroinflammation [86]. Pharmacokinetic work indicates that gastrodin and its active metabolite can be detected in blood and brain, supporting biological plausibility for central nervous system effects [87]. Experimental research on blood–brain barrier dysfunction further suggests that pathways related to endothelial protection may be relevant to postoperative cognitive injury [88]. Gastrodin has also shown neuroprotective effects in a toxin-induced Parkinson’s disease model, indicating possible anti-apoptotic and glial-modulating properties [89]. Therefore, gastrodin should be viewed as a high-priority candidate for future trials rather than as an already established perioperative neuroprotective drug.

Cognitive training offers a complementary non-pharmacological approach. Its main advantage is that it does not depend on drug exposure, anesthetic depth, or immediate emergence characteristics, which makes it attractive for elderly patients and patients at high risk of medication-related adverse effects. A cardiac-surgery meta-analysis found that cognitive training improved postoperative cognitive outcomes and quality of life, although its effect on POD was less certain [69]. Basic research also supports the concept that activity-dependent hippocampal plasticity and BDNF-related pathways can protect against cognitive decline [90]. In this context, cognitive training may be best understood as a cognitive-reserve strategy that can be combined with pharmacological interventions rather than as a substitute for optimized perioperative management.

Current evidence indicates that intranasal insulin, gastrodin, cognitive training, esketamine, and dexmedetomidine are promising interventions for preventing or alleviating postoperative delirium and postoperative cognitive dysfunction. Their effects may be mediated through multiple mechanisms, including reduction of neuroinflammation, enhancement of neuroplasticity, stabilization of perioperative brain metabolism, and protection of hippocampal function. This study used a Bayesian network meta-analysis to synthesize direct and indirect evidence from 46 randomized controlled trials involving 9,928 patients and 20 pharmacological and non-pharmacological interventions, providing a comprehensive comparative evaluation. However, several limitations should be noted. Some interventions were supported by only a small number of studies with limited sample sizes, which may reduce the precision of effect estimates and may inflate the uncertainty of rankings. In addition, heterogeneity and risk of bias existed across studies, particularly in non-pharmacological trials where blinding and allocation concealment were difficult. The MMSE network contained fewer studies, and the ranking direction depended on the definition of change values; therefore, MMSE results should be interpreted as exploratory. Moreover, most studies focused on short-term outcomes and lacked long-term follow-up. Therefore, large-scale, multicenter randomized controlled trials with rigorous designs and extended follow-up are needed to confirm these results and determine optimal intervention strategies.

## 6 Conclusion

This study systematically evaluated the efficacy of diverse pharmacological and non-pharmacological interventions for preventing and mitigating postoperative neurocognitive complications in patients undergoing cardiac surgery under general anesthesia. Under the prespecified outcome-direction coding, in which lower SUCRA values indicate greater effectiveness, intranasal insulin, gastrodin, cognitive training, esketamine, and dexmedetomidine showed promising benefits, particularly for POD and POCD outcomes. MMSE-change findings should be interpreted cautiously because they were based on fewer studies and were sensitive to change-score direction. Further high-quality, large-scale, multicenter randomized controlled trials with long-term follow-up are required to confirm these effects, assess their generalizability, and provide stronger evidence to guide optimization of anesthesia and perioperative management strategies.

## Data Availability

The original contributions presented in the study are included in the article/Supplementary material, further inquiries can be directed to the corresponding author/s.
